# Synthetic biology strategies for sustainable bioplastic production by yeasts

**DOI:** 10.71150/jm.2501022

**Published:** 2025-03-28

**Authors:** Huong-Giang Le, Yongjae Lee, Sun-Mi Lee

**Affiliations:** 1 Division of Energy and Environment Technology, University of Science and Technology (UST), Daejeon 34113, Republic of Korea; 2 Clean Energy Research Center, Korea Institute of Science and Technology (KIST), Seoul 02792, Republic of Korea; 3 Division of Environmental Science and Ecological Engineering, Korea University, Seoul 02841, Republic of Korea

**Keywords:** yeast, synthetic biology, bioplastics, PLA, PBAT, PHA

## Abstract

The increasing environmental concerns regarding conventional plastics have led to a growing demand for sustainable alternatives, such as biodegradable plastics. Yeast cell factories, specifically *Saccharomyces cerevisiae* and *Yarrowia lipolytica*, have emerged as promising platforms for bioplastic production due to their scalability, robustness, and ease of manipulation. This review highlights synthetic biology approaches aimed at developing yeast cell factories to produce key biodegradable plastics, including polylactic acid (PLA), polyhydroxyalkanoates (PHAs), and poly (butylene adipate-co-terephthalate) (PBAT). We explore recent advancements in engineered yeast strains that utilize various synthetic biology strategies, such as the incorporation of new genetic elements at the gene, pathway, and cellular system levels. The combined efforts of metabolic engineering, protein engineering, and adaptive evolution have enhanced strain efficiency and maximized product yields. Additionally, this review addresses the importance of integrating computational tools and machine learning into the Design-Build-Test-Learn cycle for strain development. This integration aims to facilitate strain development while minimizing effort and maximizing performance. However, challenges remain in improving strain robustness and scaling up industrial production processes. By combining advanced synthetic biology techniques with computational approaches, yeast cell factories hold significant potential for the sustainable and scalable production of bioplastics, thus contributing to a greener bioeconomy.

## Introduction

Ever since the revolutionary discovery of the first synthetic plastic in 1907 by Leo Baekeland and commercial production in the 1950s, the world has witnessed the unprecedented growth of the plastic industry (Perera et al., 2023). Driven by the increasing usage of plastic-based materials, global demand for plastic production is projected to reach 1.2 billion tonnes by 2060 (Jayakumar et al., 2023). Plastics are traditionally produced from non-renewable resources, such as fossil fuels, which make them inherently unsustainable and non-biodegradable materials (Liu et al., 2022). Approximately 79% of this non-biodegradable plastic waste ends up in landfills, incinerators, or waterways (Jayakumar et al., 2023), posing threats to both terrestrial and marine ecosystems, disrupting biodiversity and causing long-term environmental damage (Ali et al., 2021).

**Fig. 1.**
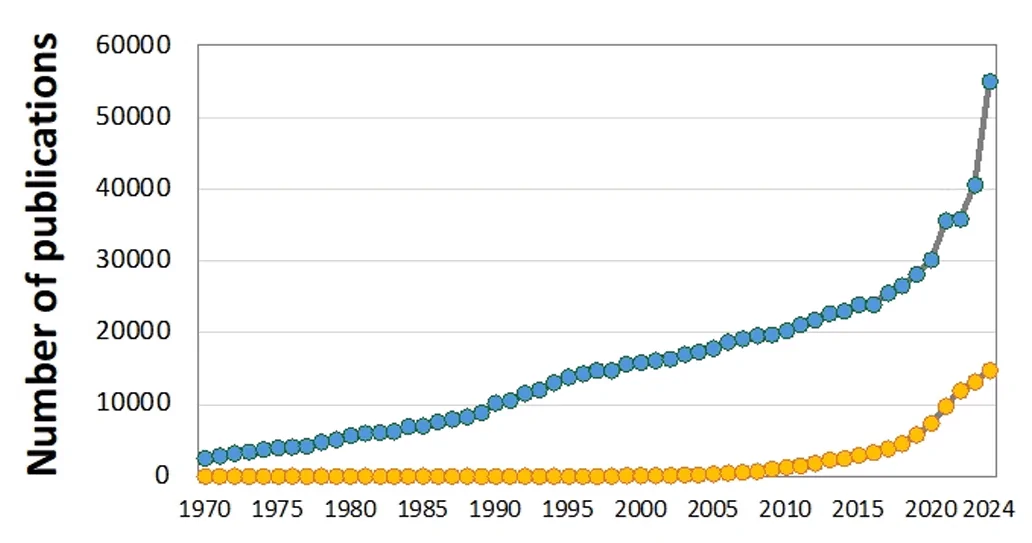
Number of research publications on bioplastics (blue) and biodegradable (yellow) plastics between 1970 and 2024. Data from the Google Scholar search engine.

**Fig. 2.**
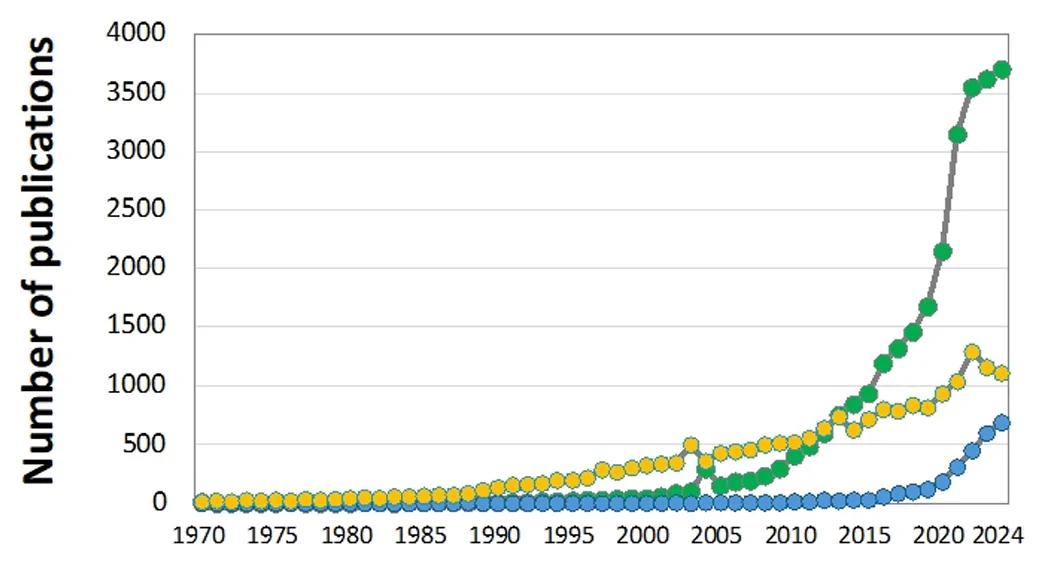
Number of research publications on PLA (green), PBAT (blue), and PHAs (yellow) between 1970 and 2024. Data from the Google Scholar search engine.

To solve these environmental issues, effort has been put into developing sustainable, biodegradable alternatives to petroleum-based non-biodegradable plastics. Bioplastics, derived from renewable resources have gained significant attention (Emadian et al., 2017) due to their reduced carbon footprint and diverse end-of-life options (Narancic et al., 2018). The term “bioplastics” encompasses bio-based and/or biodegradable plastics (Lackner et al., 2023), including PLA (polylactic acid), PHAs (polyhydroxyalkanoates), bio-PBS (polybutylene succinate), and cellulose-based plastics (Lackner et al., 2023; Moshood et al., 2022; Narancic et al., 2020). Microbial synthesis of bioplastics offers advantages such as lower production cost, milder processing conditions, and product specificities compared to chemical synthesis (Huang et al., 2021; Zhao et al., 2023). As of December 2024, searching for the keyword "microbial production of biodegradable plastics" on Google Scholar yields 202,000 results. This significant increase in research from 1970 to 2024 highlights the growing demand for bioplastics and biodegradable plastics, as illustrated in Fig. 1. Among various bioplastics, PLA, PBAT, and PHAs have garnered increasing research interest, as reflected in publication trends (Fig. 2). Their prominence is driven by their diverse applications, promising biodegradability, and compatibility with microbial production platforms.

To meet the growing demand for microbial production of these bioplastics, synthetic biology has emerged as a transformative approach for engineering microbial cell factories. Since its introduction by Eric Kool at the American Chemical Society annual meeting in 2000 (Rawls, 2000), synthetic biology has garnered significant investment from industry and widespread attention from academia (Garner, 2021). This interdisciplinary field integrates principles from biology, engineering, and computational science to design and construct biological systems with novel functions. In the context of bioplastic production, synthetic biology enables the precise engineering of microbial metabolism to optimize the synthesis of bioplastic monomers and polymers. Key synthetic biology techniques, such as CRISPR-Cas9 for genome editing, modular DNA assembly for pathway construction, adaptive laboratory evolution, and computational modeling for pathway and fermentation conditions optimization, have demonstrated their power in advancing microbial cell factory engineering. These techniques have been applied to engineer various microorganisms, including bacteria, cyanobacteria, yeast, and fungi, to improve production efficiency (Agarwal et al., 2022; Xu et al., 2025). As a result, notable achievements in microbial bioplastic production have been reported, including lactic acid production at 154 g/L in *Pichia kudriavzevii* (Park et al., 2018) and 142 g/L in *Saccharomyces cerevisiae* (Song et al., 2016), succinate at 105.8 g/L in *Actinobacillus succinogenes* (Guettler et al., 1996), muconic acid at 64.5 g/L in *Escherichia coli* (Choi et al., 2019), and PHB at 98.7 g/L in *Alcaligenes latus* (Wang & Lee, 1997).

Among these, bacteria, particularly *E. coli*, are the most amenable to genetic modifications. However, bacterial systems typically have lower tolerances to harsh industrial conditions and are prone to phage contamination and unwanted horizontal gene transfer. This poses significant issues in industrial-scale production, especially if antibiotic-resistant genes spread (Carruthers & Lee, 2022; De Souza & Gupta, 2024). Filamentous fungi, on the other hand, grow slowly and may produce aflatoxins and other by-products, raising safety concerns (Kayitesi et al., 2023; Zheng et al., 2022).

Yeast-based systems, particularly *S. cerevisiae* and *Yarrowia lipolytica*, show promise due to their scalability, robustness, and well-established genetic engineering tools. As an already proven industrial production host, *S. cerevisiae* demonstrates superior tolerance to various stress conditions, and its membrane-bound organelles facilitate complex biosynthetic pathways. It holds the generally recognized as safe (GRAS) status and is easy to handle. As the first eukaryote to have its whole genome sequenced, *S. cerevisiae* has plentiful engineering tools for genetic modification, as well as a highly efficient homologous recombination system for precise genetic modifications (Hong & Nielsen, 2012; Lian et al., 2018). These features make *S. cerevisiae* an ideal platform for the industrial production of bioplastics and their monomers. In addition to *S. cerevisiae*, *Y. lipolytica*, a non-conventional yeast, also exhibits unique properties for use as a host in bioplastic production. Its innate metabolic pathway for fatty acid metabolism benefits the production of certain bioplastic monomers. Furthermore, *Y. lipolytica* can grow under extreme conditions, such as low pH, high salt levels, and high cell density, while still producing significant quantities of valuable molecules (Larroude et al., 2018). All these qualities make *Y. lipolytica* an attractive option for industrial bioplastic production.

Despite these advantages offered by yeast, the number of studies using these eukaryotes as host cells remains limited compared to research conducted on bacteria. To promote the progress in bioplastics production utilizing these promising yeast chassis, it is essential to conduct a systematic review covering recent progress, discussing strategies, and suggesting potential solutions for current challenges. Therefore, in this review, we aim to report on the recent advancements in bioplastic production using yeast, discuss strategies and tools employed to achieve significant product titers, and suggest potential solutions for existing bottlenecks. Additionally, we explore the transformative potential of recent advancements in machine learning, artificial intelligence, and computer-aided tools in optimizing the Design-Build-Test-Learn (DBTL) cycle for improved production of bioplastics and their monomers in yeast.

### Recent progress in engineering yeasts for the production of polylactic acid (PLA)

Polylactic acid (PLA) is a biodegradable polymer made from lactic acid (LA) that serves as a sustainable alternative to petroleum-based plastics (Shekhar & Mondal, 2024). PLA is valued for its biodegradability, biocompatibility, transparency, and strong physical properties (Balasubramanian et al., 2023; Henton et al., 2005; Huang et al., 2021; Masutani & Kimura, 2018). It is used in various industries, including pharmaceuticals, food packaging, and agriculture (Casalini et al., 2019). PLA accounted for 31% of the global production capacities of bioplastics in 2023 (European-Bioplastics, 2023), and expected to replace about 10% of traditional plastics, with global demand projected at 30 million tons annually (Huang et al., 2021). Interest in PLA has spurred a significant increase in related publications over the past two decades (Mehmood et al., 2023). LA, with the molecular formula CH_3_CH (OH)COOH, is a crucial building block in chemistry. It can be produced through chemical or biological methods, with the biological method via microbial fermentation being preferred due to its cost-effectiveness and ability to produce optically pure LA (Ojo & De Smidt, 2023). This approach utilizes renewable resources like food waste and biomass, significantly reducing production costs, as raw materials comprise 40-70% of total costs (Tejayadi & Cheryan, 1995). Currently, over 90% of LA is produced through fermentation (Rodrigues et al., 2015).

While most bioplastics are not directly synthesized in nature, the monomer that makes up PLA, LA, can be produced as a by-product of the glycolytic energy cycle in various organisms, including bacteria, such as *Lactobacillus*, *Streptococcus*, *Leuconostoc* species, fungi, yeast, cyanobacteria, and algae (Huang et al., 2021). The production titer and yields of the natural producers have significantly improved through synthetic biology approaches. For example, engineered *Corynebacterium glutamicum* has been reported to produce up to 264 g/L of lactic acid using glucose as the carbon source (Tsuge et al., 2019). Additionally, recent advancements have enabled the production of polymeric PLA directly from *E. coli*, achieving a titer of 0.955 g/L (Jung et al., 2010; Shi et al., 2022).

**Fig. 3.**
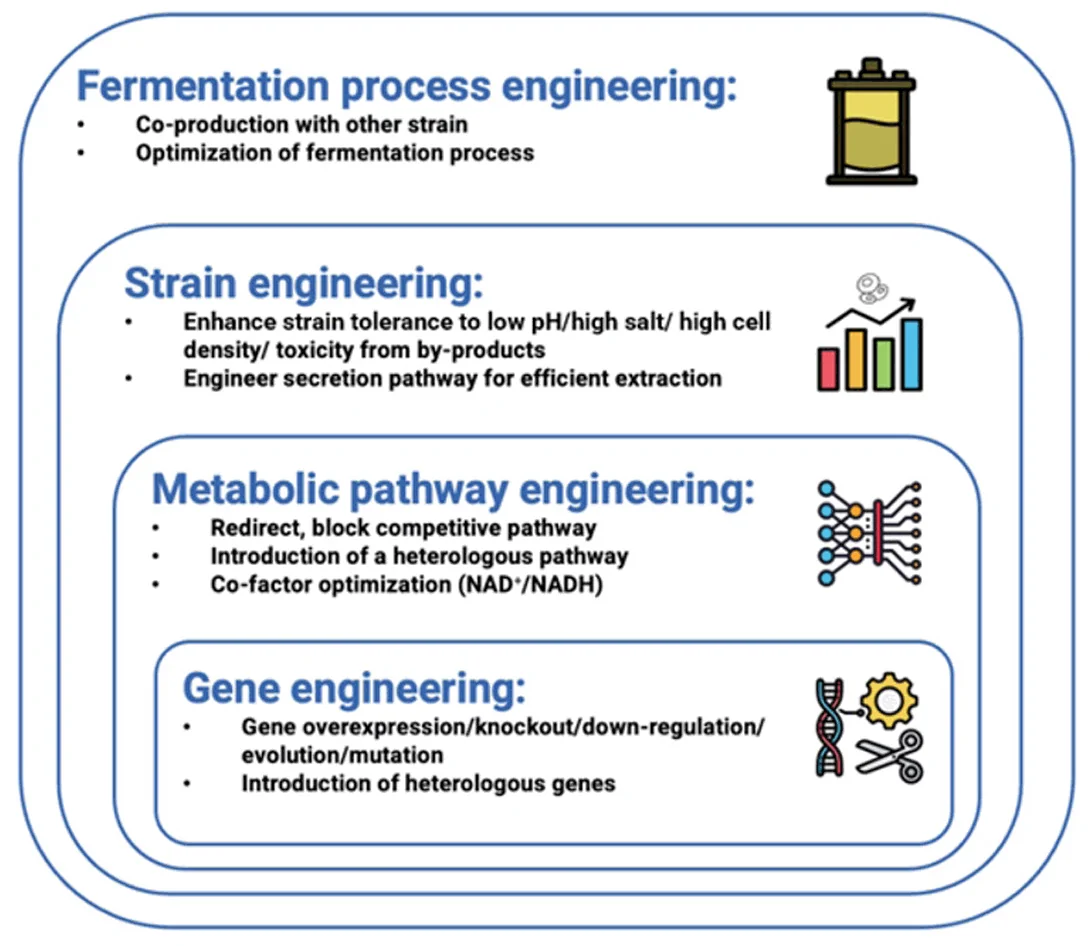
Engineering strategies for yeast chassis in the production of biodegradable plastics.

While bacterial host strains dominate in the industrial production of LA, yeast-based systems have also shown comparable efficiency in recent years. For instance, *S. cerevisiae* SP1130 strain achieved an LA titer of 142 g/L (Song et al., 2016) by expressing heterologous lactate dehydrogenase (*LDH*) genes, disrupting and attenuating competitive pathways, and introducing bacterial acetylating acetaldehyde dehydrogenase (A-ALD) genes to compensate for acetyl-CoA production. Similarly, another study reported an L-LA titer of 121.5 g/L L-LA using the same host strain, by further engineering in the metabolic pathway to rewire metabolic flux (Zhu et al., 2022). In the production of D-LA, *Pichia kudriavzevii* NG7, an isolated strain from grapes, achieved a titer of 154 g/L when its *PDC1* was replaced with a *D-LDH* from *Lactobacillus plantarum* (Park et al., 2018). Recently, *S. cerevisiae* and *Y. lipolytica* have also been explored as a production host for polymeric PLA production, though the titers remained suboptimal (Lajus et al., 2020). To achieve such advancements, various strategies have been employed, which can be classified into four main levels: gene/enzyme, metabolic pathway, strain, and fermentation process (Fig. 3).

**Fig. 4.**
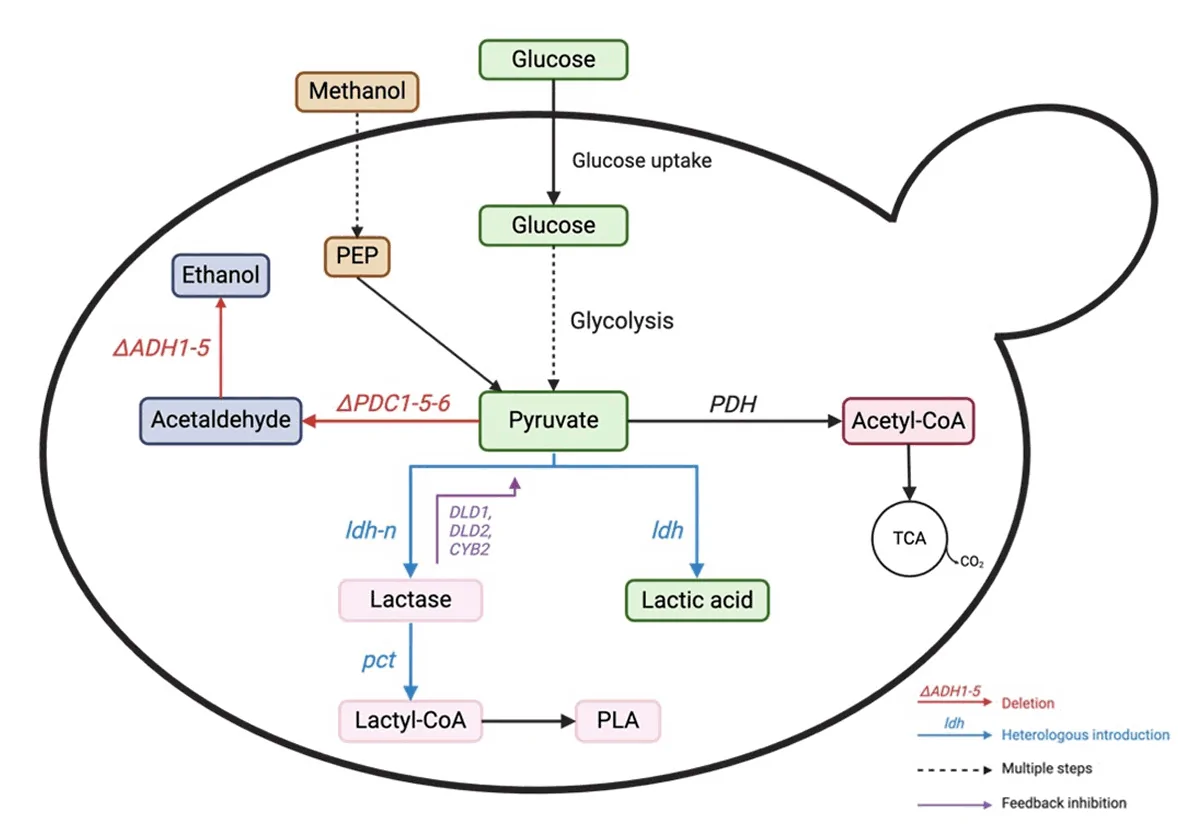
Lactic acid production pathway from glucose in yeast. *PDC*: pyruvate decarboxylase, *ADH*: alcohol dehydrogenase, *LDH*: lactate dehydrogenase, *PDH*: pyruvate dehydrogenase. The red color represents gene deletion deactivation. The blue color represents the introduction of heterologous genes/gene overexpression.

The introduction of the heterologous pathway in the non-native producers of yeast typically begins with the incorporation of genes that encode key enzymes. In a notable study, six copies of the bovine *L-LDH* (L-lactate dehydrogenase) were introduced into the genome of *S. cerevisiae*, resulting in the L-lactate titer of 122 g/L and the optical purity above 99.9% (Saitoh et al., 2005). In another study, *Candida boidinii* was engineered by disrupting the *PDC1* encoding pyruvate decarboxylase, thereby reducing ethanol production. Under optimized batch fermentation, this strain achieved 85.9 g/L of L-LA production within 48 h, with a productivity of 1.79 g/L/h (Osawa et al., 2009). The metabolic engineering strategies used to enhance LA production in yeast are summarized in Fig. 4, which illustrates the key genetic modifications, including heterologous gene expression and pathway rewiring.

Another strategy for enhancing LA production involves increasing the pool of acetyl-CoA to boost cell growth and carbon catabolism. Coupled with *LDH* expression and attenuation of competitive pathways, LA production in *S. cerevisiae* was further improved, resulting in a titer of 142 g/L with productivity of 3.55 g/L/h (Song et al., 2016). A more recent study in *Pichia pastoris* employed a combined strategy of *LDH* screening, employing NADPH-dependent LDH, blocking LA consumption pathways, and constructing a coordinated dual pathway in both cytoplasm and mitochondria. Through these strategies, the group achieved 4.2 g/L of L-LA from methanol as the sole carbon source, demonstrating the potential of methylotrophic yeasts for methanol-based biotransformation (Wu et al., 2025).

At the strain level, engineering efforts focus on enhancing the robustness of the host organism under industrial fermentation conditions while reducing the burden for the downstream processes. Although yeast generally tolerates harsher conditions better than bacteria, further improvements in tolerance are still necessary to achieve economic feasibility. To this end, adaptive laboratory evolution (ALE) and the introduction or modification of specific target genes are employed to improve strain tolerance toward acid stress, high salt, high sugar concentrations, and toxicity from by-products generated during a fermentation process. ALE employed to improve lactic acid tolerance resulted in a 17% increase in L-LA production, reaching a titer of 119 g/L using buckwheat husk hydrolysates (Jang et al., 2021). In another example, combined efforts of ALE with the introduction of a heterologous *LDH* and deletion of *ADH* and *GPD*, the genes involved in ethanol and glycerol synthesis, respectively, as well as the genes involved in the D-LA degradation pathway (*DLD1*). These efforts resulted in the production of 82.6 g/L D-LA with a yield of 0.83 g/g glucose in *S. cerevisiae* JHY5730 (Baek et al., 2017). The global metabolic engineering strategy (GMES) using a cocktail δ-integration method has proven to be effective in LA production, during which random integration of 13 genes from *Leuconostoc mesenteroides* into genomic DNA of *S. cerevisiae* yielded a D-LA titer of 33.9 g/L of even without neutralizing agents (Mitsui et al., 2020). The development of strain with high acid tolerance has also been developed for neutralizer-free fermentation process resulted in a LA titer of 74.57 g/L (Zhang et al., 2023a). At the global scale of cellular metabolism, machine-learning models have been utilized to optimize gene expression levels and thus increase metabolic rates. Yamamoto et al. constructed a yeast library with various glycolytic enzymes and *D-LDH* expressions. Data from this library were then used to build a machine-learning model that identified key genes impacting D-LA productivity and their expression levels (Yamamoto et al., 2023). Additionally, optimizing cellular redox balance has been applied as a strategy to enhance LA production. Beyond deleting genes in competitive pathways against LA production, Lee et al. (2015) deleted two genes, *nde1* and *nde2*, involved in regulating redox balance to redistribute cytosolic NADH. This modification led to a 32.6% increase in the final L-LA titer, reaching 117 g/L in a fed-batch bioreactor, and underscoring the importance of redox balance engineering (Lee et al., 2015).

Once strains were developed, fermentation process optimization could be applied to further enhance LA production. Various alternative substrates have been explored as feedstock, including cane juice (Saitoh et al., 2005), lignocellulosic biomass (Choi et al., 2024), and methanol (Wu et al., 2025; Yamada et al., 2019) to enhance both economic feasibility and sustainability in LA production. In practice, significant advancements often emerged from integrating multiple strategies at various levels. To meet industrial and economic demands, a comprehensive framework must address challenges at each stage, enabling more efficient and sustainable LA production.

Through combined efforts, yeast strains of *S. cerevisiae* and *Y. lipolytica* have been transformed to polymerase lactic acid for the production of PLA. Introduction of a polyhydroxyalkanoate (PHA) synthase (PhaC1437_Ps6-19_), propionyl-CoA transferase (Pct540C), and acetyl-CoA acetyltransferase PhaA, and acetoacetyl-CoA reductase PhaB_1_ resulted in the accumulation of 7.326 mg PLA/g-DCW (dry cell weight) in *S. cerevisiae* (Ylinen et al., 2021). In another study in *Y. lipolytica*, its native lactic acid consumption pathway was disrupted, and a heterologous PLA synthesis pathway based on bacterial propionyl-CoA transferase (PCT), which converts lactic acid into lactyl-CoA, was introduced. Following the evolution of PHA synthase, polymerizing lactyl-CoA into PDLA, the *Y. lipolytica* strain was able to produce PLA with a yield of 26 mg/g-DCW, demonstrating the potential of metabolic engineering in yeast for direct production of PLA (Lajus et al., 2020). As shown in Table 1, a comparison of lactic acid production by engineered yeast highlights the differences in yields achieved through various genetic modifications.

Despite achieving LA titers exceeding 100 g/L, engineering yeast for industrial LA production faces several challenges. Yeast lacks native biosynthetic pathways for PLA production, necessitating either the optimized heterologous expression of lactate dehydrogenase for LA production or the construction of synthetic lactyl-CoA synthesis pathway for PLA production. Redirecting pyruvate flux toward LA/PLA competes with ethanol and biomass formation, often leading to redox imbalance due to disruptions in NADH/NAD^⁺^ homeostasis. Although PLA has been produced in *Y. lipolytica*, limitations in genetic manipulation tools and low editing efficiency in non-model yeast strains prolong strain development (Gohil et al., 2017). Addressing these barriers will require integrated biosynthetic approaches, including dynamic pathway regulation and the development of new synthetic biology tools tailored for non-model yeast strains, to establish yeast as a viable platform for the commercial production of LA and PLA.

### Recent progress in engineering yeasts for the biosynthesis of Poly (butylene adipate-co-terephthalate (PBAT)

Poly (butylene adipate-co-terephthalate) (PBAT) is a biodegradable co-polyester synthesized from 1,4-butanediol (BDO), adipic acid (AA), and terephthalic acid (TPA). The molecular structure with aliphatic and aromatic units brings excellent mechanical properties for PBAT. Compared with other biodegradable polyesters such as PLA and poly (butylene-co-succinate) (PBS), PBAT expresses outstanding flexibility, higher elongation at break (close to 700%), and is fully biodegradable (Ferreira et al., 2019). These properties make PBAT a highly promising material for a wide range of applications. Currently, the predominant method for synthesizing PBAT is direct esterification using TPA or methyl terephthalate (DMT), along with AA, and BDO, which is environmentally friendly, low cost, and more efficient than the ester-exchange method (Xu, 2023).

**Fig. 5.**
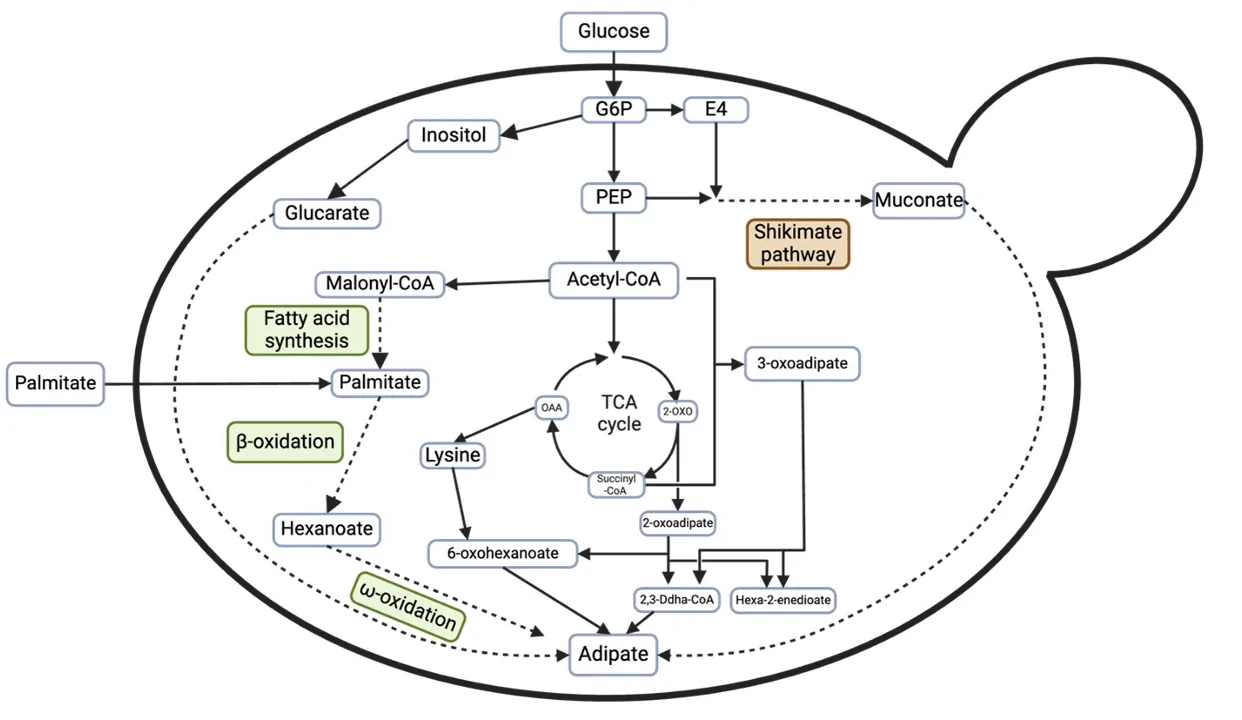
Overview of metabolic pathway to adipic acid production (Averesch et al., 2018). G6P: Glucose-6-Phosphate, E4: Erythrose-4-Phosphate, PEP: Phosphoenolpyruvate, OAA: Oxaloacetate, 2-OXO: 2-Oxoglutarate.

Adipic acid (AA), a key monomer for PBAT polymerization, is currently produced mostly from fossil-based resources. However, growing concerns over greenhouse gas emissions and the environmental impact of petrochemical processes have driven increased interest in bio-based AA production (Zhao et al., 2018). To this end, various metabolic pathways have been explored (Kruyer & Peralta-Yahya, 2017; Niu et al., 2002; Raj et al., 2018; Zhao et al., 2018), including 1) glycolysis-based pathways (via glucarate and shikimate pathways), 2) TCA cycle-based pathways (via 2-oxoadipate, 3-oxoadipate, and lysine pathways), and 3) fatty acid metabolism-based pathways (via fatty acid synthesis and fatty acid catabolic pathways) (Averesch et al., 2018) (Fig. 5). Despite extensive efforts, microbial AA production still faces numerous challenges and bottlenecks. A comprehensive study in 2018 evaluated 16 different pathways for producing AA and cis,cis-muconic acid (ccMA), a precursor of AA, in *E. coli* and *S. cerevisiae* using a novel computational tool (NExT-EMA) (Averesch et al., 2018). The study demonstrated that high theoretical AA yields are achievable in a microbial system; however, the optimal host organism, metabolic pathways, and energy transport system can vary depending on whether product yield, titers, or thermodynamic feasibility is the priority. In terms of product yields, routes via 3-oxoadipate pathway offered the highest theoretical values but were limited by a thermodynamic equilibrium on the substrate side. In contrast, shikimate pathway-based routes were thermodynamically more favorable, achieving moderate yields. For commercial-scale production, only the dehydroshikimate and isochorismate routes constructed in *E. coli* showed robust performance, delivering a carbon yield of 75% or higher and a product concentration exceeding 5 mM. However, these high yields came at the cost of lower titers, highlighting the trade-off between yield and titer in the current metabolic designs.

The highest titer of AA produced by yeast (50 g/L) was disclosed in Verdezyne Inc. patent, achieved by *Candida* spp. with engineered fatty acid catabolism (Beardslee & Picataggio, 2012). However, the specific background and metabolic engineering approaches used in that work remain unclear. In a later study, a mutant strain of *Candida tropicali* deleting *AOX* genes encoding for acyl-CoA oxidases resulted in an AA titer of 12.1 g/L using C12 methyl laurate as a substrate. Although *C. tropicalis* exhibits natural resistance to adipic acid and can efficiently utilize oil and fatty acids (Skoog et al., 2018), its industrial potential is constrained by its pathogenic nature, which poses risks to human health (O’Brien et al., 2021). Therefore, the development of alternative, safer host strains is crucial for large-scale AA production. A recent study manipulated the β-oxidation pathway in *Y. lipolytica*, by disrupting *POX1* and *POX3* to prevent adipic acid AA oxidation and enhanced ω-oxidation through overexpression of cytochrome P450 monooxygenase (*ALK5*), and reductase (*CPR1*), and fatty alcohol oxidase (*FAO1*) (Lee et al., 2024). Together with medium optimization in a two-stage bioconversion process, they achieved an AA titer of 1.18 g/L, representing a 9.7-fold increase over the parental strain. Though yeast has demonstrated potential for AA production, its titers remain lower than those reported for *E. coli*. The highest AA titer in *E. coli* was reported to be 68 g/L by harnessing the *Thermobifida fusca*-derived reverse adipate degradation pathway (Tfu-RADP), together with the elimination of the succinate-CoA ligase gene (Zhao et al., 2018). A comparison of adipic acid produced by various strains, including strategies, tools, culture conditions, and titers, is summarized in Table 2.

**Fig. 6.**
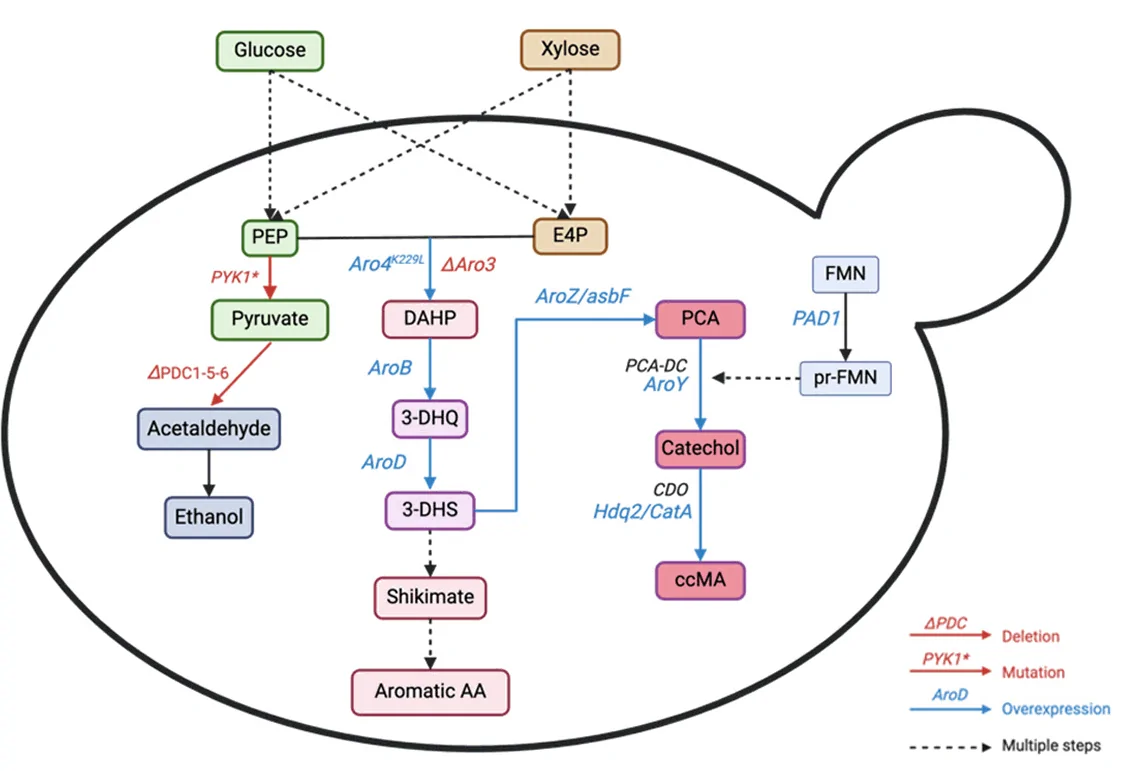
Schematic representation of the metabolic pathway to produce muconic acid. PEP: Phosphoenolpyruvate, DAHP: 3-deoxyarabinoheptulosonate 7-phosphate, 3-DHQ: 3-dehydrogenate, 3-DHS: 3-DHS: 3-dehydroshikimate, PCA: protocatechuic acid, PCA-DC: PCA decarboxylase, CDO: catechol 1,2-dioxygenase, FMN, flavin mononucleotide, prFMN, prenylated FMN. The red color represents gene deletion deactivation. The blue color represents the introduction of heterologous genes/gene overexpression.

One promising strategy for AA production is to synthesize its precursor, cis,cis-muconic acid (ccMA), which can be efficiently converted into AA through chemo-catalytic hydrogenation with a conversion yield exceeding 97% (Niu et al., 2002; Polen et al., 2013). Moreover, ccMA itself is a versatile platform chemical that can serve as a building block for various high-value compounds (Cachera et al., 2024). As shown in Fig. 6, ccMA is typically synthesized by the conversion of glucose into phosphoenolpyruvate (PEP) and erythrose 4-phosphate (E4P), which feed enter the shikimic acid pathway, a route for synthesizing aromatic amino acids (Wu et al., 2022). The specific genetic modification and pathways required to optimize ccMA production remain an active area of research. For instance, intermediates such as 3-dehydroshikimate (DHS) and chorismite (CSA) can be supplied by introducing heterologous pathways (Yang et al., 2024). Several studies have re-engineered the shikimate pathway for ccMA production (Choi et al., 2020). The highest titer reported of ccMA (64.5 g/L) was achieved by *E. coli* AB2834 strain (Choi et al., 2019).

In recent years, the ccMA production in yeast has been significantly improved (Table 3). Nicolai et al. introduced a heterologous MA synthesis pathway and deleted the genes involved in ethanol production along with eliminating feedback inhibition in the shikimate pathway. These efforts resulted in the production of 4.5 g/L ccMA by *S. cerevisiae* (Nicolai et al., 2021). In a following study by Tönjes et al. (2024) a higher titer of MA production (9.3 g/L) was achieved by reducing C2 dependency through the deletion of PDC genes and minimizing the accumulation of the intermediate protocatechuic acid (PCA) by *PAD1* overexpression. Later, a similar carbon-flux redirection strategy was employed by overexpression and mutation of several genes (*PYK1*, *PYC1*, *PCK1*, *ScARO4^K229L^*, *EcaroB*, *EcaroD*) in *S. cerevisiae*, achieving 22.5 g/L of ccMA production (Wang et al., 2022). Recently, 108 publicly accessible and non-conventional yeast strains have been screened for their tolerance to adipic acid and other dicarboxylic acids (Pyne et al., 2023). As a result, *Pichia occidentalis*, exhibiting tolerance to 20 g/L adipic acid, was selected and successfully engineered, resulting in a ccMA titer of 38.8 g/L with a yield of 0.134 g/g glucose. This yield surpasses all previously reported data from *S. cerevisiae*, highlighting the potential of non-conventional yeast as a viable host strain for ccMA and AA production.

Low product titers in AA and ccMA production are largely attributed to yeast’s sensitivity to the pathway intermediate, catechol, which inhibits growth at 0.5 mM compared to 5 mM in *Pseudomonas*. Additionally, protocatechuic acid decarboxylase (AroY) exhibits 90% lower activity in yeast than in bacteria due to improper flavin cofactor incorporation at 30°C (Marshall et al., 2017). Endogenous aldehyde dehydrogenases in *S. cerevisiae* divert carbon flux by converting adipate precursors to hexanoic acid, accounting for 35% of the carbon flux. Although the deletion of *ALD6* improved yields, it reduced growth rates by 55% due to NAD^+^ depletion (Ning et al., 2022). Addressing these challenges will require integrated synthetic biology approaches, including enzyme optimization, cofactor regulation, and strain engineering, to enhance yeast tolerance to catechol, improve PCA decarboxylase activity, and minimize byproduct formation for efficient production of AA and ccMA.

### Recent progress in yeast engineering for the biosynthesis of Polyhydroxy-alkanoates (PHAs)

Polyhydroxyalkanoates (PHA) are biobased and biodegradable polyesters produced by diverse microorganisms (Chen, 2010). They have drawn considerable attention as sustainable alternative to traditional fossil-based polymers (Rigouin et al., 2019) as evidenced by over 612 identified patent families (Elvers et al., 2016) and more than 100,000 Google patents results (as of 2024). Globally, PHAs represents 4.8% of the total bioplastics production in 2023, with projections indicating an increase to 13.5% by 2028, second to PLA (European-Bioplastics, 2023).

PHA is biosynthesized by various microorganisms (Chen, 2010) under nutrient limited conditions (Saharan et al., 2014). PHA consists of varying numbers of carbon atoms, 3-, 4-, 5-, and 6-hydroxycarboxylic acids (Zhang et al., 2023b). Based on the number of carbon atoms in the alkyl side chain of the monomers, PHA is categorized into short-chain-length hydroxyalkanoic acids (sclPHA) with up to two carbon atoms, and medium-chain-length hydroxyalkanoic acids (mclPHA) with three or more carbon atoms (Hazer & Steinbuchel, 2007). Although hydroxyalkanoate monomers rarely occur independently, the polymeric forms possess properties determined by their constituent monomers (Chen, 2010; Zhou, 2022). For instance, polyhydroxybutyrate (PHB), the simplest sclPHA, is brittle, whereas mclPHA exhibits a range of properties from rubber-like elasticity to high heat-resistant (Pollet & Avérous, 2011).

**Fig. 7.**
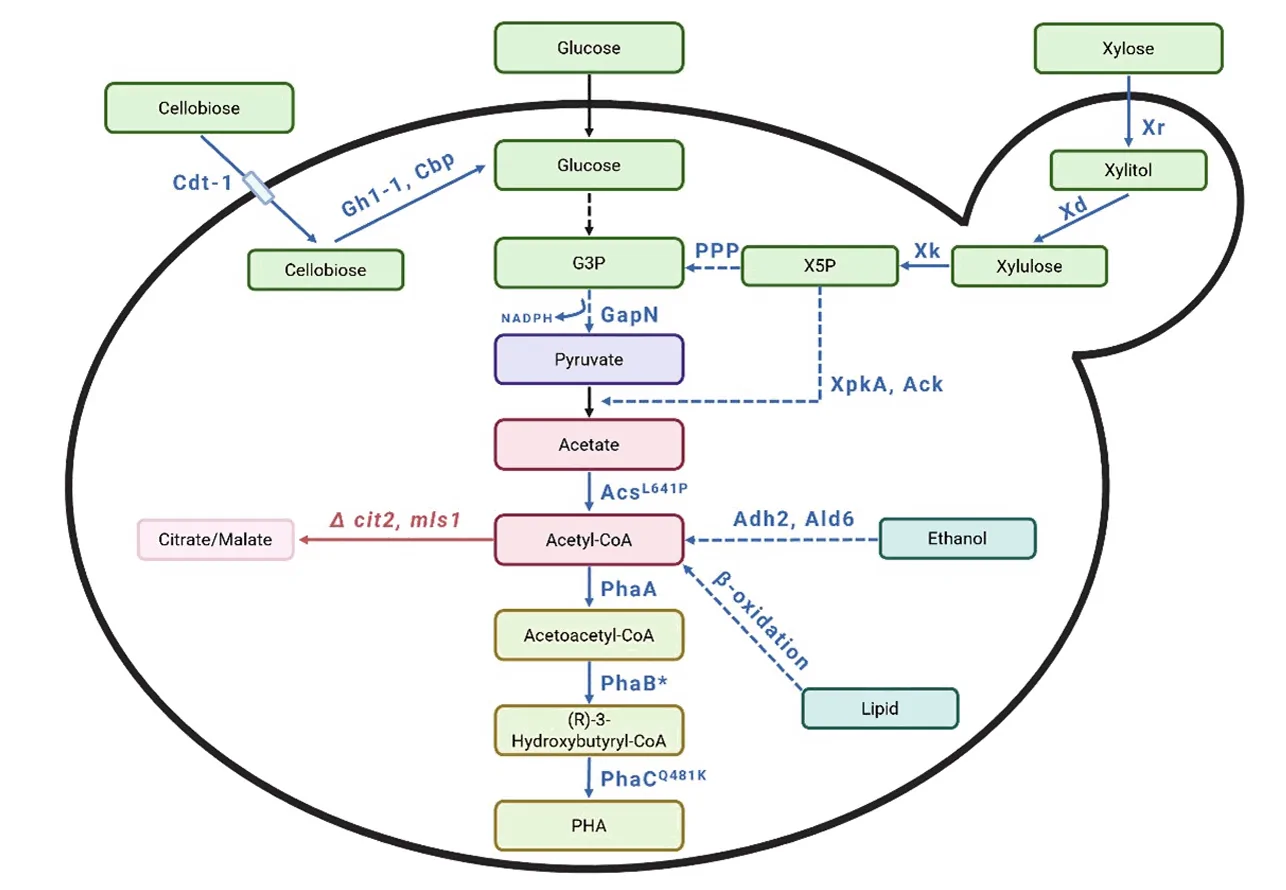
Schematic representation of the metabolic pathway to produce polyhydroxyalkanoates from various substrates. G3P: Glyceraldehyde-3-phospahate, X5P: Xylulose-5-phosphate, PPP: Pentose phosphate pathway, Cdt-1: cellodextrin transporter, Gh1-1: β-glucosidase, Cbp: cellobiose phosphorylase, Xr: xylose reductase, Xd: xylitol dehydrogenase, Xk: xylulokinase, XpkA: xylulose-5-phosphate phosphoketolase, Ack: acetate kinase, GapN: NADP^+^-dependent glyceraldehyde-3-phosphate dehydrogenase, Adh2: alcohol dehydrogenase, Ald6: acetaldehyde dehydrogenase, *cit2*: citrate synthase, *mls1*: malate synthase PhaA: acetyl-CoA acetyltransferase, PhaB: acetoacetyl-CoA reductase, PhaC: PHA synthase. The red color represents gene deletion or deactivation. The blue color represents the introduction of heterologous genes or gene overexpression. A solid arrow indicates that the reaction occurs in a single step, while a dashed arrow indicates that it occurs over multiple steps.

The known metabolic pathways for PHA biosynthesis include the 3-hydroxypropionate (3-HP) cycle, the glycerol-3-phosphate (G3P) pathway, and the β-oxidation pathway (Subramanian et al., 2024) (Fig. 7). Of these, the G3P pathway is recognized as a key route for PHA synthesis in various organisms. Core enzymes in the G3P pathway include acetyl-CoA acetyltransferase (PhaA), acetoacetyl-CoA reductase (PhaB), and polyhydroxyalkanoate synthase (PhaC), typically originating from organisms such as *Cupriavidus necator* (formerly known as *Rastonia eutropha*) and *Azotobacter vinelandii* (Subramanian et al., 2024). Notably, PhaC plays a pivotal role in PHA polymerization (Chek et al., 2019), while phasin proteins (PhaP) facilitate stable accumulation of PHA granules in the cytoplasm (Brown et al., 2022; Chek et al., 2019).

The first reported case of PHA production using yeast involved the heterologous expression of the *phaC* gene from Alcaligenes eutrophus in *S. cerevisiae* (Leaf et al., 1996). Since then, various studies have focused on manipulating *S. cerevisiae* as a production host for PHA. These efforts include diversifying substrates to include pentoses such as xylose (Sandström et al., 2015) and lignocellulose biomass (Ylinen et al., 2022) and engineering the strain to co-produce PHA alongside other products such as ethanol or D-LA (Tran et al., 2023; Ylinen et al., 2021). Beyond *S. cerevisiae*, research has explored the use of non-conventional yeasts such as *Y. lipolytica*. Since the early 2000s, efforts to industrialize *Y. lipolytica* have intensified, and the development of synthetic biology tools has led to a sharp increase in related publications (Park & Ledesma-Amaro, 2023). The robust fatty acid metabolism can significantly enhance PHA synthesis (Ledesma-Amaro & Nicaud, 2016). Furthermore, unlike *S. cerevisiae*, which possesses only a single acyl-CoA oxidase (*AOX*), *Y. lipolytica* has six AOX isozymes with different substrate specificities, providing additional flexibility in β-oxidation pathway. During β-oxidation pathway, (R)-3-hydroxy-acyl-CoA is generated, serve as a precursor for PHA synthesis (Gao et al., 2015). Several studies have demonstrated increased PHA yields by expressing PhaC to convert fatty acids into PHA or modulating the β-oxidation pathway to direct fatty acid flux toward PHA precursors, successfully producing 1.11 g/L PHA (Gao et al., 2015; Haddouche et al., 2011).

Research on utilizing yeast as a PHA synthesis factory frequently relies on synthetic biology tools, including CRISPR-Cas9 (Larroude et al., 2018; Ylinen et al., 2022). For instance, the *phaA*, *phaB1*, and *phaC1* were integrated into the genome of *S. cerevisiae* using CRISPR-Cas9, achieving a yield of 49.4 mg/g using the lignocellulosic disaccharide cellobiose as the substrate (Ylinen et al., 2022). In this study, the metabolism of cellobiose was enabled by introducing β-glucosidase (Gh1-1), cellobiose phosphorylase (Cpb), and cellodextrin transporter (Cdt-1) genes from Neurospora crassa (Ylinen et al., 2022). Similarly, *phaA*, phaB, and *phaC* were integrated into the genome of *S. cerevisiae* as a single polycistronic expression cassette, achieving a titer of 40.3 mg/L PHB (Tran et al., 2023).

Optimizing enzyme activity represents another key strategy for enhancing PHA production. For example, PhaB from *C. necator* was replaced with NADH-dependent PhaB from purple sulfur bacterium *Allochromatium vinosum*, leading to increased PHA production (Muñoz de Las Heras et al., 2016). In this study, the mutant strain TMB4425, containing PhaB from *A. vinosum*, achieved titers of 178 mg/L under aerobic conditions, 252 mg/L under oxygen-limiting conditions, and 360 mg/L under anaerobic conditions. These values were found to be up to 8 times higher than the strain expressing PhaB from *C. necator* (Muñoz de Las Heras et al., 2016). Furthermore, using the same TMB4425 strain, a study revealed that nitrogen limitation influences PHB production. Unlike bacteria, *S. cerevisiae* exhibited higher PHB production under nitrogen-rich conditions, achieving a PHB titer of 730 mg/L under a nitrogen-rich and oxygen-limited condition (Portugal-Nunes et al., 2017). In a recent study, site-directed mutagenesis of PhaC, replacing glutamic acid at position 130 with aspartic acid, resulted in a PHA accumulation of 28% DCW (Rigouin et al., 2019). Other studies have also identified specific amino acid residues in PhaC that are critical for PHA synthesis (Chen et al., 2014; Matsumoto et al., 2005; Rigouin et al., 2019). These studies provided a basis for enzyme design by identifying amino acid residues critical for enzyme activity, thereby enhancing PHA productivity. The detailed information on PHA production by engineered yeasts highlights the differences in yields achieved through various genetic modifications (Table 4).

Despite the relatively limited research on PHA production in yeast, which likely contributes to its currently low performance, yeast-based PHA production showed promising results. In addition to establishing synthetic pathways using codon optimization and synthetic promoter-terminator systems, advanced synthetic biology approaches, such as computational design, can be leveraged to further improve production efficiencies.

## Future Perspectives

Synthetic biology has enabled significant progress in developing microbial cell factories. Particularly, the Design-Build-Test-Learn (DBTL) cycle has emerged as a crucial paradigm, playing a significant role in the efficient and automated construction of microbial strains (Gurdo et al., 2023). Through DBTL cycle, strain engineering can be streamlined and scaled up, especially for complex metabolic pathways involved in the production of various bioplastics such as PLA, PHA, and PBAT. Recent advancements in computer-aided synthetic biology have led to faster and more efficient strain development (Chen et al., 2020). One of the key components of DBTL cycle is Machine Learning (ML), which leverages big data to design innovative metabolic pathways and engineer strain more effectively. A primary database supporting ML-based strain development is genome-scale metabolic models. GEMs compile and analyze information on genes, proteins, enzymes, and metabolic pathways, thereby enabling predictive insight into cellular function. As ML results continue to expand GEM datasets, prediction accuracy is further improved (Liao et al., 2022). Consequently, by analyzing and learning from large databases, GEMs and ML facilitate faster and more rational decision-making during the Design and Learning phases in DBTL cycle (Liao et al., 2022). Various ML algorithms have been developed and tailored to specific research goals, underscoring their growing importance in this field (Kim et al., 2020).

Metabolic engineering of microorganisms plays a critical role in producing various chemicals and materials (Lee et al., 2012). It enables the production of desired native and non-native chemicals through microbial metabolic pathways (Na et al., 2010). Metabolic engineering generally follows two main approaches: 1) introducing synthetic pathways into microorganisms to produce chemicals that are not naturally synthesized, or 2) modifying or enhancing existing biosynthetic pathways in organisms of interest (Dasgupta et al., 2020). Synthetic biology techniques, including incorporating enzymes from various organisms into synthetic pathways, have been widely applied in traditional model organisms such as *E. coli* (Alonso-Gutierrez et al., 2013; McKenna & Nielsen, 2011; Murakami et al., 2015; Xue et al., 2013; Zhao et al., 2013). Significant achievements have also been reported in *S. cerevisiae*, a well-established model yeast, for the production of chemicals. For instance, modulation of a fatty acid biosynthesis pathway and a subsequent converting pathway of triacylglycerol (TAG) to free fatty acid along with promoter engineering harboring Kozak sequence of AAACA resulted in a free fatty acid titer of 400 mg/L (Runguphan & Keasling, 2014). Additionally, an engineering strategy that optimized oxidative succinate production of succinate via a partial TCA cycle enabled succinate production of 3.62 g/L without causing serious growth defects (Raab et al., 2010).

Similar synthetic biological approaches are increasingly applied to yeast-based bioplastic production. For example, an *in silico* study optimizing LA production in *S. cerevisiae* highlights the integration of synthetic biology and ML approaches. Researchers used the Saccharomyces Genome Database (SGD) (https://www.yeastgenome.org/) and literature reviews to predict optimal strain conditions based on correlation of glucose uptake and LA yield (Amaradio et al., 2023). They further reported increased LA production by combining gene knockouts related to carbon and oxygen utilization with culture medium optimization (Amaradio et al., 2023).

As ML algorithms continue to mature and biological databases grow, synthetic biological research will likely expand from DNA sequence-level predictions to three-dimensional protein modeling and the estimation of optimal culture conditions (Feehan et al., 2021). These innovations can significantly reduce the time and resources needed for introducing synthetic metabolic pathways and optimizing native ones, thus enhancing the economic feasibility of bioplastic production. Consequently, employing large-scale data processing and computer modeling such as GEM-based ML algorithms will become a cornerstone of DBTL cycles, providing the fundamental technological framework for a sustainable bioplastic industry (Liao et al., 2022).

## Conclusion

The growing concerns about environmental problems have led to the demand for sustainable production of biodegradable plastics to replace fossil-based counterparts. Among various microbial platforms, yeast-based systems exhibit unique properties and advantages over bacteria, making them promising hosts for the industrial production of biodegradable plastics. In this review, we summarize the latest advancements in yeast-based biosynthesis of biodegradable plastics and their monomers, alongside the strategies, tools, and metabolic pathways that have enabled these developments. Despite significant progress over the past decade, yeast cell factories required further improvement, including improving strain tolerance, pathway optimization, and downstream process scalability. To face these challenges, the integration of advanced computational tools, particularly computer-aided design, holds significant potential to solve for overcoming current bottlenecks. Innovations in computational methods would significantly enhance the efficiency and effectiveness in DBTL cycle to develop the powerful strains for biodegradable plastics production. Furthermore, while not covered in detail here, downstream processes such as product recovery and purification, often reliant on extensive trial-and-error type optimization, could also benefit from machine learning. As AI and machine learning continue to advance rapidly, these innovations are expected to revolutionize biotechnology, facilitating breakthroughs and promoting more efficient and sustainable production systems.

As AI and machine learning continue to evolve, their integration with synthetic biology and process engineering is expected to revolutionize bioplastic production. These technologies enable precise, automated, and scalable bioprocess development, significantly enhancing the economic viability and sustainability of yeast-based biodegradable plastics. Continued interdisciplinary research will be essential to fully realize these innovations, paving the way for more efficient and commercially feasible bioplastic production systems.

## Figures and Tables

**Table 1.** Comparison of lactic acid production by engineered yeasts

Strains	Substrate	Engineering Strategies	Tools	Culture condition	Titer (g/L)	Productivity (g/L/h)	References
*Saccharomyces cerevisiae* SP1130	Glucose	Express heterologous lactate dehydrogenase (*LDH*) genes, attenuating several key pathway genes including glycerol-3-phosphate dehydrogenase 1 (*GPD1*) and cytochrome-c oxidoreductase 2 (*CYB2*). Knocking out the pyruvate decarboxylase 1 (*PDC1*) and alcohol dehydrogenase 1 (*ADH1*) to disrupt the ethanol production pathway, redirect metabolic flux towards lactic acid production. Introduce an alternative pathway to produce acetyl-CoA using the acetylating acetaldehyde dehydrogenase (*A-ALD*) gene from *E. coli* to compensate for the attenuated *ADH1*	Heterologous gene expression, Introduction of a synthetic pathway	Fed-batch fermentation	142	3.55	Song et al. (2016)
*S. cerevisiae* T165R	Cane juice	Integrate six copies of *L-LDH* into the genome of host cell	Multi-integration of a heterologous gene	1-L jar fermenter	L-LA 122	-	Saitoh et al. (2005)
*S. cerevisiae* S.c-NO.2-100	Glucose	Rewire the biosynthetic pathway for LA production by screening and introducing *L-LDH*, reducing ethanol accumulation by deleting *PDC1*, *ADH1*, introducing bacterial acetyl-CoA synthesis pathway. Strain evolution for acid tolerance by adaptive evolution. Delete NADH-consuming enzymes (NDE1/2) to enhance the cofactor availability. Overexpress bacterial transporters *JEN1* and remodel the cell membrane by deleting *SAM2* for efficient LA export	Heterologous gene expression, Introduction of heterologous transporter, ALE	5-L batch bioreactor	L-LA 121.5	1.69	Zhu et al. (2022)
*S. cerevisiae* BK01	Acetate-rich buckwheat husk hydrolysates	Adaptive laboratory evolution (ALE) on 8% lactic acid on *S. cerevisiae* SR8LDH strain which was previously constructed by the introduction of LDH gene from *Lactobacillus acidophilus* ATCC4356 under the control of the PGK1 promoter. The mutant BK01 was characterized by genome sequencing and metabolomic profiling	ALE, metabolomic profiling	20 mL fermentation	L-LA 119	1.24	Jang et al. (2021)
*S. cerevisiae* ASc- d789M	Glucose	Screen *D-LDH* from bacteria, overexpress *LpDLDH*, delete glycerol production genes *GPD1*, *GPD2*, and lactate dehydrogenase gene *DLD1* to minimize by-products, downregulation *ADH1* using the L-methionine repressible promoter to minimize the impact on growth. Created an intraspecific hybrid strain by mating the engineered strain with a weak acid-tolerant wild-type strain BCC39850 to improve acid tolerance and D-LA production	Gene knockout by CRISPR/Cas9, CRISPR/Cas12a, yeast mating	Fed-batch fermentation	D-LA 23.41 ± 1.65	0.81	Watcharawipas et al. (2021)
*S. cerevisiae*	Glucose	Genome evolution applied a method combining GMES and CRISPR-δ integration (GMES/CRISPR) to integrate 13 bacterial genes including* HXT7*, *HXK2*, *PGI1*, *PFK1*, *PFK2*, *FBA1*, *TPI1*, *TDH3*, *PGK1*, *GPM1*, *ENO2*, and *PYK2*, and d-lactate dehydrogenase (d*-LDH*) into genomic DNA	Genome evolution by GMES and CRISPR-δ integration, overlap extension PCR, transcriptome analysis, real-time PCR	Semi-neutralizing fermentation	D-LA 52.2	2.17	Mitsui et al. (2020)
*S. cerevisiae* JHY5330	Glucose	Introduce D-lactate dehydrogenase gene (*ldhA*) from bacteria, reduce D-LA consumption by deleting *DLD1* encoding D-lactate dehydrogenase and *JEN1* encoding monocarboxylate transporter, eliminate ethanol production gene *PDC1* and *ADH1*, glycerol production genes *GPD1* and *GPD2*, improve strain LA tolerance by ALE and overexpression of *HAA1* encoding a transcriptional activator involved in weak acid stress response	ALE, gene knockout, PCR-mediated method using the Cre/loxP system, quantitative reverse transcription PCR (qRT-PCR)	Fed-batch fermentation	D-LA 112	2.2	Baek et al. (2016)
*S. cerevisiae* JHY5610	Glucose	Employed ALE for LA-tolerance of host strain, coupled with introduction of LA production gene *Lm. ldhA,* and deletion of ethanol and glycerol production genes (*ADH*, *GPD*) and D-LA degradation genes to redirect metabolic flux toward LA production	ALE, gene knockout, whole genome sequencing	Fed-batch fermentation	D-LA 82.6	1.5	Baek et al. (2017)
*S. cerevisiae*	Glucose	Rewire metabolic fluxes toward the production of L-lactic acid by deleting *PDC1*, *CYB2*, *GPD1*, and replacing *LDH*. Re-engineer the intracellular redox balance by deleting *NDE1* and *NDE2* encoding isoenzymes of the external NADH dehydrogenase	Gene knockout, re-engineer intracellular redox	Fed-batch	L-LA 117	-	Lee et al. (2015)
*Pichia pastoris*	Methanol	Screen LDH genes, co-utilize LDH cofactors, knockout *DLD1*, *DLD2* and *CYB2* to block LA consumption pathway, and construct a coordinated dual pathway in cytoplasm and mitochondria	Site-directed mutation, CRISPR-Cas9, fluorescence microscopy analysis	Fed-batch fermentation	L-LA 4.2	0.0126	Wu et al. (2025)
*Pichia kudriavzevii* NG7	Glucose	Replace the pyruvate decarboxylase 1 gene (*PDC1*) with the d-lactate dehydrogenase gene (*d-LDH*) derived from *Lactobacillus plantarum*, improve strain tolerance by ALE	ALE, whole genome sequencing	Bioreactor fed-batch	D-LA 154	4.16	Park et al. (2018)
*Candida boidinii*	Glucose	Disrupted *PDC1* to reduce ethanol production, introduce and express Bovine L-LDH gene under the *PDC1* promoter and optimize fermentation condition	Heterologous gene expression	Batch fermentation	L-LA 85.9	1.79	Osawa et al. (2009)
*S. cerevisiae*	Glucose	Poly (D-lactic acid) (PDLA) and copolymer P (LA-3HB) were produced by express PHA synthase PhaC1437_Ps6-19_, propionyl-CoA transferase Pct540Cp, acetyl-CoA acetyltransferase PhaA, and acetoacetyl-CoA reductase PhaB1	Gibson Assembly, modular cloning method (MoClo), GC-MS, NMR analysis	Erlenmeyer baffled flasks fermentation	PDLA 0.73% (CDW)	-	Ylinen et al. (2021)
*Yarrowia lipolytica*	Glucose, racemic lactic acid	Disrupt lactic acid consumption pathway, introduce heterologous pathway for PDLA production containing propionyl-CoA transferase (pct) from *Clostridium propionicum* to convert lactic acid into lactyl-CoA, and an evolved polyhydroxyalkanoic acid PHA synthase polymerizing lactyl-CoA into PDLA	Site-directed mutagenesis, UHPLC-FTMS	Erlenmeyer baffled flasks fermentation	PDLA 26 mg/g-DCW	-	Lajus et al. (2020)

**Table 2.** Comparison of adipic acid production by engineered yeast

Strains	Substrate	Engineering Strategies	Tools	Culture condition	Titer	References
*E. coli* BL21 (DE3)	Glycerol	Reconstruct five-step reverse adipate-degradation pathway (RADP) from *Thermobifida fusca* in *E. coli*, then overexpress 5-Carboxy-2-pentenoyl-CoA reductase (*Tfu_*1647) - the gene coding for the rate-limiting step in the RADP, delete succinate-CoA ligase gene (*sucD*) to eliminate competitive	Introduction of a synthetic pathway, Gibson assembly, CRISPR/Cas9, gene overexpression, SDS-PAGE	Fed-batch fermentation in 5-L bioreactor	68.0 g/L	Zhao et al. (2018)
*Candida* spp.	Fatty acid	Rewire the ω- then the β-oxidation pathway	-	Two-stage fed-batch process	50 g/L	Beardslee & Picataggio (2012)
*Candida tropicalis*	C12 methyl laurate	Create a mutant strain of *C. tropicali* by deleting *AOX* genes encoding acyl-CoA oxidases to produce AA via ω- and the β-oxidation pathway	Gene deletion	Bioreactor, fed-batch	12.1 g/L	Ju et al. (2020)
*S. cerevisiae*	Glucose	Use the reverse adipate degradation pathway (RADP) from *Thermobifida fusca*, co-expressing genes of Tfu_ 0875, Tfu_2399, Tfu_0067, Tfu_1647, Tfu_2576, and Tfu_ 2576 in *S. cerevisiae*	Introduction of a synthetic pathway, Gibson assembly, overlap extension PCR	Fed-batch bioreactor	10.09 mg/L	Zhang et al. (2020)
*S. cerevisiae*	Glucose	Rewire the cis, cis-muconic acid pathway by expressing enoate reductases (ERs) from *Bacillus coagulans* to convert ccMA into AA	Introduction of a synthetic pathway, Gibson assembly	Three stage fermentations	2.59 mg/L, ccMA > 284 mg/L	Raj et al. (2018)
*Y. lipolytica*	Fatty acid methyl esters	Regulate the β-oxidation pathway by disrupting acyl-CoA oxidases (*POX1* and *POX3*) and enhance ω-oxidation through overexpression of *ALK5*, *CPR1*, and *FAO1*, two-phase bioprocess to enhance AA production	Gene deletion, two-phase bioprocess	20 ml in 250 ml baffled flask	1176.2 mg/L	Lee et al. (2024)

**Table 3.** Comparison of cis,cis-muconic acid production by engineered yeast

Strains	Substrate	Engineering Strategies	Tools	Culture condition	Titer (g/L)	Productivity (g/L/h)	References
*S. cerevisiae*	Glucose	Employed ccMA-biosensor, coupled with GFP expression to screen UV-mutagenesis libraries for ccMA producing yeast, then overexpress genes encoding for PCA decarboxylase and AROM protein and restore URA3 prototrophy.	UV-mutagenesis, fluorescence activated cell sorting (FACS), heterologous gene expression	Fed-batch	20.8	0.139	Wang et al. (2020)
*S. cerevisiae*	Glucose	Rewiring the shikimate pathway flux and enhancing the phosphoenolpyruvate supply by mutating *PYK1* (A336S) to limit PEP-to-pyruvate conversion and overexpressing *PYC1* and *PCK1* to redirect pyruvate to PEP, overexpress DAHP synthase (*ScARO4^K229L^*), 3-dehydroquinate synthase (*EcaroB)*, and 3-dehydroquinase	Cas9-assisted approach, Gibson assembly, USER cloning, EasyClone method	2L fermenter	22.5	0.19	Wang et al. (2022)
*S. cerevisiae*	Glucose, xylose, mixture	Eliminated feedback inhibition in the shikimate pathway, insert heterologous pathway for MA production using 3-dehydroshikimate (DHS), protocatechuic acid decarboxylase (PCAD) and oxygen-consuming catechol 1,2-dioxygenase (CDO), delete PDC to eliminate ethanol production, minimized PCA production by enhancing PCAD overexpression and production of its co-factor	Introduction of a synthetic pathway, genome editing using CRISPR/Cas9	300 ml shake flasks with 40 mL YP medium	4.5	-	Nicolai et al. (2021)
*S. cerevisiae*	Glucose and xylose	Eliminates C2 dependency and ethanol production by ALE and deleting *PDC1*, *PDC5*, and *PDC6,* improving MA tolerance for host strain, promoting growth and production of target product by internal deletion of *MTH1*, *MTH1*^ΔM41-T78^, overexpressing *PAD1* as well as reduces side production of intermediate protocatechuic acid (PCA)	Introduction of a synthetic pathway, enzyme engineering, ALE	Fed-batch	9.3	0.100	Tönjes et al. (2024)
*S. cerevisiae*	Glucose	Combine adaptive laboratory evolution (ALE) and rational metabolic engineering, improve flux by truncation of ARO1 and overexpress an endogenous aromatic decarboxylase	ALE, Gibson assembly	Fed-batch bioreactor	2.1	-	Leavitt et al. (2017)
*S. cerevisiae*	Glucose	Employ the computational tool YEASTRACT for predicting novel transcriptional repressors and OptForce strain-design for identifying non-intuitive pathway interventions, strating from glycolytic and pentose phosphate pathway pathway, follow by engineer *ARO2*, *ARO3*, *ARO4*, and the pentafunctional *ARO,* delete *ric1* to increase transcription of* ARO2*, *ARO3*, *ARO4*	Computational tool for predicting engineering target, Introduction of a synthetic pathway Gibson assembly	-	0.32	-	Suástegui et al. (2017)
*S. cerevisiae*	Glucose, xylose (supplement 1 g/L catechol)	Overexpress xylose isomerase gene from *Bacteroides valgutus* and pentose phosphate pathway genes from *S. cerevisiae*, then imported a three step ccMA production pathway from *E. coli* with *AroZ*-Neu, *AroY*-Com, and *CatA*-Cup genes, further overexpression of gene *Aro1* (with a stop codon of AroE) and feedback-resistant Aro4opt mutant gene, ALE to improve xylose fermentation and ccMA	Introduction of a synthetic pathway, Enzyme engineering, ALE	Batch, shake flask	0.424 (1.286 with catechol supplemented)	-	Liu et al. (2020)
*S. cerevisiae*	Glucose, supplement amino acids	Balance MA pathway performance with aromatic amino acid prototrophy by destabilizing Aro1, delete *ARO4* and *ARO3,* introduce AroZ, AroY, Hqd2 CatA, Pad1.	CRISPR-mediated homology-directed repair (HDR)	Fed-batch bioreactor	5.1	-	Pyne et al. (2018)
*S. cerevisiae*	Glucose	Construct cassettes using USER cloning-ligation-PCR, express a three-step heterologous pathway using *KpAroY.B*,* KpAroY.Ciso, CaCatA,* engineering *TKL1, ZWF1, Aro1AroEΔ, Aro4^K229L^*	Introduction of a synthetic pathway, CRISPR/Cas9, in vivo recombination USER assembly and RNA interference	Batch, shake flask	0.8	-	Kildegaard et al. (2019)
*Pichia occidentalis*	Glucose	Screen host strain with high tolerance to MA, develop genome editing toolkit to engineer the non-conventional yeast and introduce the heterologous pathway for ccMA production	CRISPR-Cas9, antibiotic marker recycling, high-throughput screening	Fed-batch	38.8	0.511	Pyne et al. (2023)

**Table 4.** Comparison of PHA production by engineered yeast

Strains	Substrate	Engineering Strategies	Tools	Culture condition	Titer (mg/L)	Yield (mg/g)	References
*E. coli*	Glucose, xylose	Knocked out ndh gene. Introduced arabinose/xylose transport proteins from *Bacillus subtilis* (*xylA*, *xylB*, and *araE*). Incorporated the PHB biosynthesis pathway from *C. necator*	Modification of ribosome binding sites	Fed-batch culture	PHB 21010	-	Huo et al. (2017)
*S. cerevisiae*	Silver grass, glucose, xylose	Constructed a polycistronic PHB cassette with *phaA*, *phaB*, and *phaC* using 2A peptide sequences. Introduced Cas9-expressing plasmid, gRNA, and polycistronic PHB cassette into *S. cerevisiae* via electroporation. Integrated the polycistronic PHB cassette into the genome	CRISPR-Cas9 mediated genome editing, 2A peptide sequence integration	Serum bottle, microaerobic	PHB 40.3	0.93 mg/g silver grass	Tran et al. (2023)
*S. cerevisiae*	Glucose	Expressed XpkA and Ack from *Aspergillus nidulans* to increase acetyl-CoA levels. Incorporated an acetyl-CoA synthase variant (Acs^L641P^) from *Salmonella enterica*. Integrated GapN (NADP⁺-dependent glyceraldehyde-3-phosphate dehydrogenase) from *Streptococcus mutans* into the genome via the Li-Ac method. Codon optimized all genes for efficient expression	Codon optimization DNA 2.0 and GenScript for synthesizing genes, P*_TEF1_*-P*_PGK1_* bidirectional promoter	Shake flask, anaerobic	PHB 180	0.03 mg/g glucose	Kocharin et al. (2013)
*S. cerevisiae*	Glucose	Synthesized *phaA*, *phaB*, and *phaC* based on *R. eutropha* H16. Introduced them into *S. cerevisiae* in plasmid form using the Li-Ac method. Used the strain described by Chen et al. (2012) to increase acetyl-CoA levels. Deleted *cit2* and *mls1*, overexpressed *ALD6* and *ADH2*. Codon optimized all genes for efficient expression	Codon optimization, P*_TEF1_*-P*_PGK1_* bidirectional promoter, DNA 2.0 for synthesize genes	Shake flask, aerobic	PHB 43.11	0.13 mg/g glucose	Kocharin et al. (2012)
*S. cerevisiae*	Xylose	Introduced XR and XD genes to enable xylose utilization. PCR-amplified *phaA*, *phaB*, and *phaC* genes from *C. necator*. Assembled these genes into a single plasmid using In-Fusion cloning. Introduced the plasmid into *S. cerevisiae* via the Li-Ac method. Codon optimized all genes for efficient expression	In-fusion cloning, Codon optimization, overlap extension PCR	Shake flask, aerobic	PHB 45	1.17 mg/g xylose	Sandström et al. (2015)
*S. cerevisiae*	Xylose	Achieved PHB production under anaerobic conditions using xylose as the carbon source. Replaced PhaB from *C. necator* with NADH-dependent PhaB from *Allochromatium vinosum*. Introduced the gene into *S. cerevisiae* in plasmid form via the Li-Ac method	Codon optimization, heterologous gene expression, promoter and terminator pairing	Bioreactor, anaerobic	PHB 360	7 mg/g xylose	Muñoz de Las Heras et al. (2016)
*S. cerevisiae*	Xylose high nitrogen supply	Investigated whether nitrogen limitation in yeast influences PHB production. Introduced Xr and Xd from *Scheffersomyces stipitis* to enable xylose utilization. Replaced PhaB from *C. necator* with NADH-dependent PhaB from *A. vinosum*. Assembled these genes into a single plasmid using In-Fusion cloning. Introduced the plasmid into *S. cerevisiae* via the Li-Ac method. Codon optimized and PCR-amplified all genes	In-fusion cloning, Codon optimization, heterologous gene expression, promoter and terminator pairing	Shake flask, anaerobic	PHB 730	13.8 mg/g xylose	Portugal-Nunes et al. (2017)
*S. cerevisiae*	Cellobiose	Integrated *phaA*, *phaB1*, and *phaC1* into the *S. cerevisiae* genome using Gibson assembly, Li-Ac method, and CRISPR-Cas9. Inserted Gh1-1 from *Neurospora crassa* and Cbp from *Ruminococcus flavefaciens* for cellobiose metabolism using the Modular Cloning (MoClo) approach. Codon optimized all genes for efficient expression	Gibson Assembly, Modular cloningCRISPR-Cas9 mediated genome integration, promoter and terminator pairing	Bioreactor	PHB -	49.4 mg/g cellobiose	Ylinen et al. (2022)
*Y. lipolytica*	Tridecanoate	Directed carbon flux in *Y. lipolytica* toward either the β-oxidation pathway or the PHA biosynthesis pathway based on the genotype of *POX1-6* genes encoding acyl-CoA oxidase isoenzymes. Downregulated the R-3-hydroxyacyl-CoA dehydrogenase multifunctional enzyme domain in the fatty acid β-oxidation pathway via site-directed mutagenesis, generating the MFE-A^G16S^BC variant. Deleted an acyltransferase gene and overexpressed enoyl-CoA hydratase 2 to further redirect fatty acids from lipid synthesis to the β-oxidation pathway	site-directed mutagenesis, *loxP*-Cre recombinase, pGEMT easy cloning	Shake flask	PHA -	7.3% DCW	Haddouche et al. (2011)
*Y. lipolytica*	Tridecanoate	Directed carbon flux in *Y. lipolytica* toward either the β-oxidation pathway or the PHA biosynthesis pathway based on the genotype of *POX1-6* genes encoding acyl-CoA oxidase isoenzymes. Introduced PhaC from *Pseudomonas aeruginosa* and modified its C-terminal region to include the 34-amino acid sequence from *Brassica napus* glyoxysomal isocitrate lyase (*icl*) for peroxisome targeting	Site-directed mutagenesis, STADEN package for sequence analysis	Shake flask	PHA -	17.64 mg/g biomass	Haddouche et al. (2010)
*Y. lipolytica*	Methyl laurate	Generated various PhaC variants through site-directed mutagenesis. Compared their production yields of mcl-PHA with different chain lengths	Site-directed mutagenesis	-	PHA -	28% DCW	Rigouin et al. (2019)
*Y. lipolytica*	Triolein	Directed carbon flux in *Y. lipolytica* toward either the β-oxidation pathway or the PHA biosynthesis pathway based on the genotype of *POX1-6* genes encoding acyl-CoA oxidase isoenzymes. Heterologously expressed PhaC1 from *Pseudomonas aeruginosa* PAO1 with a peroxisomal signal. Introduced the gene into *S. cerevisiae* in plasmid form via the Li-Ac method. Codon optimized all genes for efficient expression	Codon optimization	Shake flask	PHA 1110	5.0% DCW	Gao et al. (2015)
